# A Bayesian Approach for the Use of Athlete Performance Data Within Anti-doping

**DOI:** 10.3389/fphys.2018.00884

**Published:** 2018-07-19

**Authors:** Silvia Montagna, James Hopker

**Affiliations:** ^1^ESOMAS Department, University of Torino, Torino, Italy; ^2^Endurance Research Group, School of Sport and Exercise Sciences, University of Kent, Medway, United Kingdom

**Keywords:** anti-doping, monitoring, target testing, data analytics, competition results

## Abstract

The World Anti-doping Agency currently collates the results of all doping tests for athletes involved in elite sporting competition with the aim of improving the fight against doping. Existing anti-doping strategies involve either the direct detection of use of banned substances, or abnormal variation in metabolites or biological markers related to their use. As the aim of any doping regime is to enhance athlete competitive performance, it is interesting to consider whether performance data could be used within the fight against doping. In this regard, the identification of unexpected increases in athlete performance could be used as a trigger for their closer scrutiny via a targeted anti-doping testing programme. This study proposes a Bayesian framework for the development of an “athlete performance passport” and documents some initial findings and limitations of such an approach. The Bayesian model was retrospectively applied to the competitive results of 1,115 shot put athletes from 1975 to 2016 in order establish the interindividual variability of intraindividual performance in order to create individualized career performance trajectories for a large number of presumed clean athletes. Data from athletes convicted for doping violations (3.69% of the sample) was used to assess the predictive performance of the Bayesian framework with a probit model. Results demonstrate the ability to detect performance differences (~1 m) between doped and presumed clean athletes, and achieves good predictive performance of doping status (i.e., doped vs. non-doped) with a high area under the curve (AUC = 0.97). However, the model prediction of doping status was driven by the correct classification of presume non-doped athletes, misclassifying doped athletes as non-doped. This lack of sensitivity is likely due to the need to accommodate additional longitudinal covariates (e.g., aging and seasonality effects) potentially affecting performance into the framework. Further research is needed in order to increase the framework structure and improve its accuracy and sensitivity.

## 1. Introduction

Over the last decade the fight against doping in sports has evolved from purely biochemical testing for specific substances, to longitudinal profiling of specific biomarkers in the form of the athlete biological passport (ABP). The ABP uses longitudinal blood tests or steroid profiles, also known as modules, to indirectly demonstrate use of banned substances via a mathematical probabilistic approach (Sottas et al., 2008). This longitudinal approach has improved the efficiency of targeted testing and resulted in an increased number of positive erythropoiesis stimulating agent doping cases in recent years (Zorzoli et al., 2014). Moreover, other anti-doping initiatives as outlined in the WADA technical document for sport specific analysis, signifies a move toward a more forensic intelligence led anti-doping system, which gathers broader sources of information to inform the planning of doping tests (WADA, 2016).

A major source of information that is not currently used within anti-doping practice is athlete performance. As the goal of most doping regimes is to improve athlete performance to gain an unfair advantage in competition, it seems logical to assume that the effect of doping might be identified through the evolution of performance of an athlete over time. For example, on a global perspective, (Schumacher and Pottgiesser, 2009) demonstrate marked improvements in world best performances in male 5,000 and 10,000 m running performances following the commercial introduction of recombinant erythropoietin in the 1990s. Conversely, they also highlight a down turn in female world best discus performances following the introduction of out-of-competition anti-doping tests for anabolic steroids in the late 1980s. Therefore, it is possible to question whether longitudinal tracking of athlete performance might provide additional information (alongside haematological and steroidal ABP modules) that can be used as part of the intelligence gathering process to inform anti-doping organization's testing programmes.

The main objective of performance profiling in the form of an athlete performance passport (APP), would be to track individual performance over time in order to identify unexpected or disproportionate increases in performance that might be indicative of doping (Hopker et al., 2018). However, analysis of performance data of this nature is complex as it is difficult to differentiate between physiological increases in performance arising from normal training and/or maturation from unphysiological improvements caused by doping. Moreover, excellent athletic performance within a competition in itself is not proof of any wrong doing, or doping. Nevertheless, an APP may be useful in strengthening the sensitivity and applicability of the current ABP in the fight against doping by providing information to trigger targeted anti-doping tests for specific athletes (Iljukov et al., 2018).

In order to assess the feasibility of an APP approach, this study explores the important issue of modeling and analyzing the relationship between doping status and athlete performance accounting for some of the other covariates that impact on performance. Specifically, as a first step toward an APP, this study aims to characterize trajectories in athlete shot put performance results, while simultaneously accounting for sex and age. To address these aims, we used a Bayesian latent factor model for functional data (Montagna et al., 2012) characterizing the curve for each athlete as a linear combination of a high-dimensional set of basic functions, and placed a sparse latent factor regression model on the basis coefficients. Within this framework, it is possible to study the dependence of the curve shapes on covariates incorporated through the distribution of the latent factors, and regress a scalar response (e.g., the doping status) on a functional covariate (e.g., an athlete shot put performance curve).

## 2. Methodology

Our goal is predicting an athlete's doping status (a scalar response) given his/her shot put performance results and other non-functional covariates (e.g., age and sex). In section 2.1, we will investigate how the Bayesian latent factor regression model of (Montagna et al., 2012) can be used to represent an athlete's shot put trajectory, and in section 2.2 we will explore the extension of this method to the joint modeling of the functional predictor and the scalar outcome. Finally, in section 2.3 we will briefly discuss the advantages of a Bayesian inferential approach over classical methods.

### 2.1. Functional latent factors representation for shot put performance

**Figure 1:**
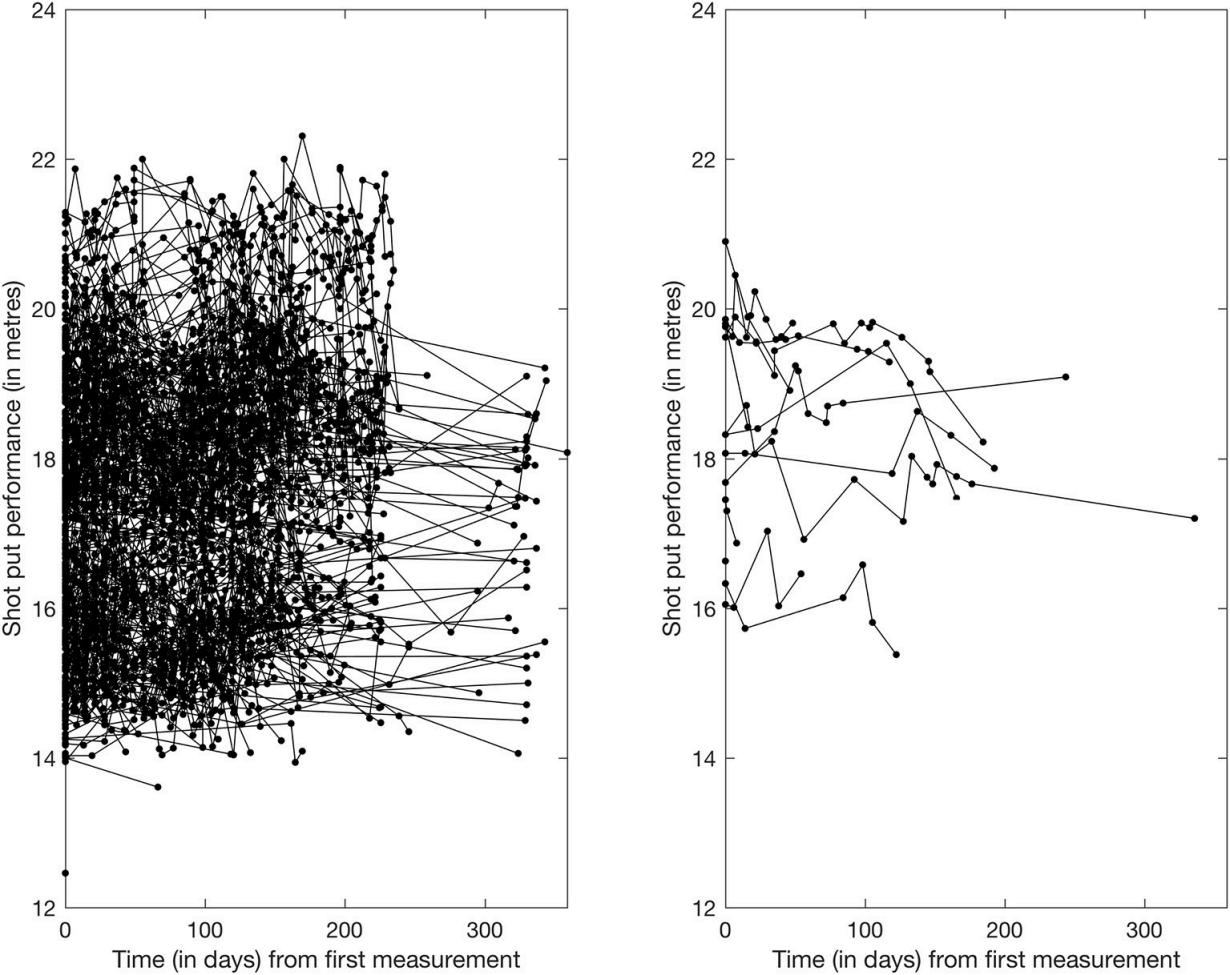
Shotput Performance trajectory of all “non-doped” (left panel) and “doped" (right panel) athletes with results in the 2012 season.

Let *n* denote the number of athletes in the study. We supposed that shot put performance results for athlete *i* are available as noisy measurements of an underlying smooth curve *f*_*i*_(*t*) at *n*_*i*_ time points *t*_*ij*_, *j* = 1, …, *n*_*i*_. Here *t*_*ij*_ denotes the time (in days) from the first measurement available for athlete *i*, thus *t*_*i*1_ = 0 for every *i*. We denote the shot put result at *t*_*ij*_ as *y*_*ij*_ and model:

(1) $$\begin{array}{r} y_{ij}=f_{i}\left(t_{ij}\right)+\epsilon_{ij}, \end{array}$$

with $\epsilon_{ij}\sim N\left(0,\psi^{2}\right)$, independently across *i* and *j*. Figure 1 shows the true shotput performance results for athletes who competed in the year of 2012. For the modeling of the random underlying smooth curves *f*_1_, …, *f*_*n*_, we follow (Montagna et al., 2012) and write ${\left\{f_{i}\right\}}_{i=1}^{n}$ in terms of a collection of fixed basis functions:

(2) $$\begin{array}{r} f_{i}\left(t_{ij}\right)=\overset{p}{\underset{m=1}{\sum }}\theta_{im}b_{m}\left(t_{ij}\right)=\text{b}{\left(t_{ij}\right)}^{⊤}\theta_{i}. \end{array}$$

It is important to use a sufficiently large *p* and to choose locally concentrated basis elements so that a rich variety of shapes for *f*_*i*_ are entertained. Hereafter, ${\left\{b_{m}\left(\cdot \right)\right\}}_{m=1}^{p}$ are chosen to be 2D isotropic Gaussian kernels:

(3) $$\begin{array}{r} b_{1}\left(t\right)=1,\;\text{and}\;b_{m}\left(t\right)=\exp\left(-b||t-\phi_{m}{||}^{2}\right),m=2,\ldots ,p, \end{array}$$

with kernel locations ${\left\{\phi_{m}\right\}}_{m=2}^{p}$ and bandwidth *b* to be specified according to prior knowledge.

The basis coefficients vectors **θ**_1_, …, **θ**_*n*_ capture all athlete-to-athlete variations in shot put performance curves. However, these vectors have a large dimension *p* and are non-sparse. To obtain a low dimensional representation of the shot put curves, we place a sparse latent factor model (Arminger, 1998) on the basis coefficients:

(4) $$\begin{array}{r} \theta_{i}=\Lambda \eta_{i}+\zeta_{i},\;\text{with}\;\zeta_{i}\sim N_{p}\left(0,\Sigma \right), \end{array}$$

where **Λ** = {λ_*ml*_} is a *p* × *k* factor loading matrix with *k* ≪ *p*, $\eta_{i}={\left(\eta_{i1},\ldots ,\eta_{ik}\right)}^{⊤}$ is a vector of latent factors for athlete *i* and $\zeta_{i}={\left(\zeta_{i1},\ldots ,\zeta_{ip}\right)}^{⊤}$ is a residual vector that is independent with the other variables in the model and is normally distributed with mean zero and a diagonal covariance matrix $\Sigma =\left(\sigma_{1}^{2},\ldots ,\sigma_{p}^{2}\right)$. The low-dimensional vectors **η**_1_, …, **η**_*n*_ are used in all subsequent parts of the model where one seeks to link the shot put curves *f*_1_, …, *f*_*n*_ with other variables of interest.

Information on covariates **x**_*i*_ (e.g., sex and age) available for athlete *i* can be incorporated though a simple linear model on the latent factors:

(5) $$\begin{array}{r} \eta_{i}=\beta^{⊤}{\text{x}}_{i}+\Delta_{i},\;\Delta_{i}\sim N_{k}\left(0,\text{I}\right), \end{array}$$

where **β** is a *r* × *k* matrix of covariates coefficients, with *r* denoting the number of covariates. We remark that the current formulation of model (1)–(5) cannot accommodate covariates changing over time, thus **x**_*i*_ can only be a vector of static covariates. For a more detailed discussion on the properties of this model, we defer to (Montagna et al., 2012).

### 2.2. Prediction of doping status

Our goal is to use the shot put performance trajectory modeled through Equations (1)-(5) to predict the doping status of an athlete *z*_*i*_. Let *z*_*i*_ = 1 if athlete *i* is convicted for doping offences at any point in his/her career, and *z*_*i*_ = 0 otherwise. From a statistical perspective, *z*_*i*_ is a Bernoulli random variable *z*_*i*_ ~ Bernoulli(*p*_*i*_), where *p*_*i*_ is the probability that athlete *i* will use doping at any point in his/her career. We follow (Montagna et al., 2012) and model *p*_*i*_ as:

(6) $$\begin{array}{r} p_{i}=\text{P}\left(z_{i}=1|\alpha ,\gamma ,\eta_{i}\right)=\Phi \left(\alpha +\gamma^{⊤}\eta_{i}\right), \end{array}$$

where Φ(·) denotes the standard normal distribution function, α is an intercept, and **γ** is a vector of unknown regression coefficients. We remark that the same set of latent factors **η**_*i*_ impacts on the shot put performance curve via the basis coefficients **θ**_*i*_ and on the response variable (the doping status) via the probability of doping. We remark that Equation (6) defines a probit model for doping status.

### 2.3. The bayesian inferential framework

**Figure 2:**
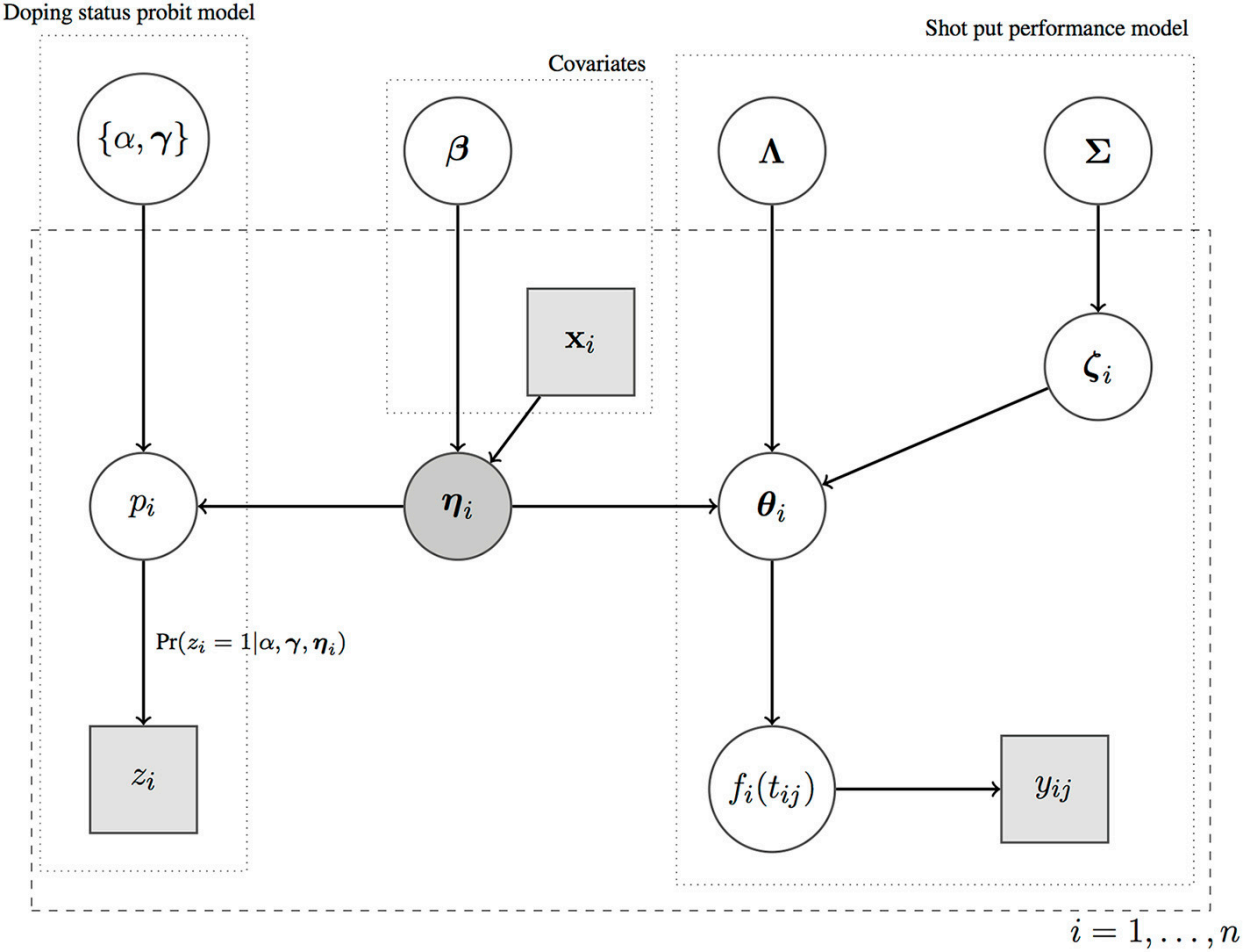
A graphical representation of the Bayesian Framework outlined in Equations (1)–(6).

To summarize our methodology, we provide a graphical representation of the model outlined in Equations (1)–(6) in Figure 2. The vector of latent factors plays the key role in linking the two component models for doping status and shot put performance curves, and the doping status *z*_*i*_ is conditionally independent of all nodes in the model given the latent factors **η**_*i*_. All parameters located outside of the dashed rectangle (α, **γ**, **β**, **Λ**, **Σ**) are shared by all athletes (parameters not indexed by *i*), and they are estimated by pulling information (*borrowing of information*) across all athletes. This is of crucial importance especially when data are sparse. If covariate information is available, covariates impact on the **η**_*i*_'s via a linear regression model.

All model parameters, which are represented by circles in Figure 2, need to be estimated from the data. To estimate these parameters, one can either proceed via classical methods or by embedding model (1)–(6) in a Bayesian framework. In classical methods, the model parameters are unknown, but fixed constants. In Bayesian statistics, the model parameters are considered to be random variables and are therefore assigned *prior* probability distributions. The priors are then combined with the likelihood to obtain the *posterior* distributions of the model parameters.

Bayesian methods and classical methods both have advantages and disadvantages, and there are some similarities. When the sample size is large, Bayesian inference often provides results for parametric models that are very similar to the results produced by classical methods. A Bayesian analysis provides a convenient setting to a wide range of models, such as the hierarchical model outlined in Equations (1)–(6). Existing numerical methods make computations tractable for virtually all parametric models. Most importantly, Bayesian inference provides a natural and principled way of combining prior experts information with the data, within a solid decision theoretical framework. For example, let us look at the intercept parameter α in Equation (6). This parameter represents the baseline risk for an athlete to use doping at some point in his/her career, regardless of athlete-specific factors (e.g., sex, age, performance). While it is impossible to know the exact proportion of doped athletes in a given sport, experts may have a sense for what this proportion (or a plausible range for this proportion) may be based on their knowledge. Suppose that experts believe that about 1% of athletes use doping. Such belief can be embedded in the prior for α, which is generally chosen to be Normal for computational convenience. Thus, α will be given a α ~ *N*(Φ^−1^(0.01), 1) prior so that the hyperprior mean is chosen to correspond to the experts knowledge (the variance can also be suitably adjusted). It is naturally possible that different people will produce different priors, and by trying different prior choices we can investigate how sensitive conclusions are to these choices. Unfortunately, it is not possible to make use of prior knowledge within a classical inferential framework.

For all the reasons above, we decide to follow the lead in (Montagna et al., 2012) and embed model (1)–(6) in a Bayesian framework by choosing prior distributions for all model parameters. Given the high dimensionality of the problem (large *n*, the number of subjects, and large *p*, the number of basis functions in Equation 2) it is practically important to choose conditionally *conjugate* prior distributions for parameters ψ^2^, α, **Λ**, **Σ**, **β**, **γ**. Conjugacy ensures that the posterior distribution of the parameters is of the same type of the prior (e.g., a Normal prior combined with a Normal likelihood as in Equation (1) returns a Normal posterior distribution), and this leads to efficient posterior computation via Markov Chain Monte Carlo. We adopt the same conjugate priors of (Montagna et al., 2012) and details on posterior computation, together with the MATLAB code to run the analysis, are available in (Montagna et al., 2012).

Despite the simplicity of this hierarchical model, the resulting model on the smooth shot put trajectories *f*_1_, …, *f*_*n*_ allows a flexible accommodation of covariate information. In particular, these curves are independent Gaussian processes with covariate dependent mean functions $𝔼\left[f_{i}\left(t\right)\right]=\overset{k}{\underset{m=1}{\sum }}\beta_{m}^{⊤}{\text{x}}_{i}{\tilde{\phi }}_{m}\left(t\right)$ and a common covariance function $\mathbb{C}\text{ov}\left\{f_{i}\left(t\right),f_{i}\left(s\right)\right\}=\overset{k}{\underset{m=1}{\sum }}{\tilde{\phi }}_{m}\left(t\right){\tilde{\phi }}_{m}\left(s\right)+\overset{p}{\underset{l=1}{\sum }}\sigma_{l}^{2}b_{l}\left(t\right)b_{l}\left(s\right)$, where **β**_*m*_ denotes the *m*th column of **β** and ${\tilde{\phi }}_{m}\left(t\right)=\overset{p}{\underset{l=1}{\sum }}\lambda_{lm}b_{l}\left(t\right)$. In summary, our model accommodates scalar (doping)-on-function (shot put trajectories) regression and the distribution of the curves is allowed to change flexibly with (static) predictors.

## 3. Data

Following Institutional ethical approval (Prop_72_2017_18) athletes data to be included within the model was obtained with permission from an open results database (www.tilastopaja.eu). Specifically, 56,000 results of elite shot put competitions of 1,115 athletes from 1976 to 2016, inclusive, were collected. Sampled data included the athlete name, IAAF ID number, date of birth, sex, country of birth, event, result in meters, finishing position, and any doping violation during the athlete's career.

Hereafter, we focus on the analysis of data collected from 2012. This enables us to use both sex and age as predictors of shot put performance. We remark that the model could be applied to all the available data from 1976 to 2016, but we would be no longer able to use age (now a longitudinal predictor) as covariate. Elite shot put performance data were collected on 352 athletes between 5^*th*^ January and 29^*th*^ December, 2012, for a total of 3290 measurements. The average number of measurements per athlete was 9.34, ranging from a minimum of 1 to a maximum of 27 measurements. The average age of the athletes was 23 years, with the youngest athlete being 15 years old and the oldest athlete being 43 years old, and 175 athletes were males. Only 13 athletes in this dataset were convicted for doping offences (*z*_*i*_ = 1), representing 3.69% of the sample. We applied model (1)–(6) to the data, and results are presented in the next section.

## 4. Results

**Figure 3:**
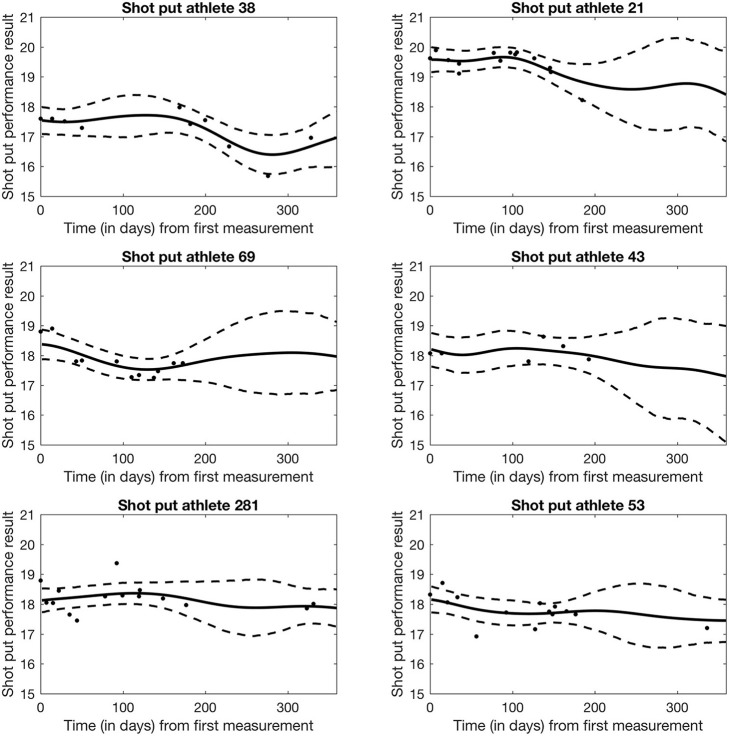
Bayesian Network Model of Shot Put performance for 6 randomly selected athletes. Solid line denotes the mean performance trend modeled from the athlete's individual data. Dashed line represents the 95% credible performance interval for the athlete.

Figure 3 shows the estimated trajectories ${\hat{f}}_{i}$ for six randomly selected athletes. As expected, we note that 95% credible intervals expand when no measurements are available, and this effect is particularly evident toward the end of the year for athletes 21, 69, and 43. Wider bands denotes higher uncertainty in the estimates due to lack of data. Left panels show three randomly selected non-doped athletes, and right panels show three randomly selected doped athletes. No significant differences emerge in the shape of the estimated trajectories at this level of analysis between doped and non-doped athletes.

**Figure 4:**
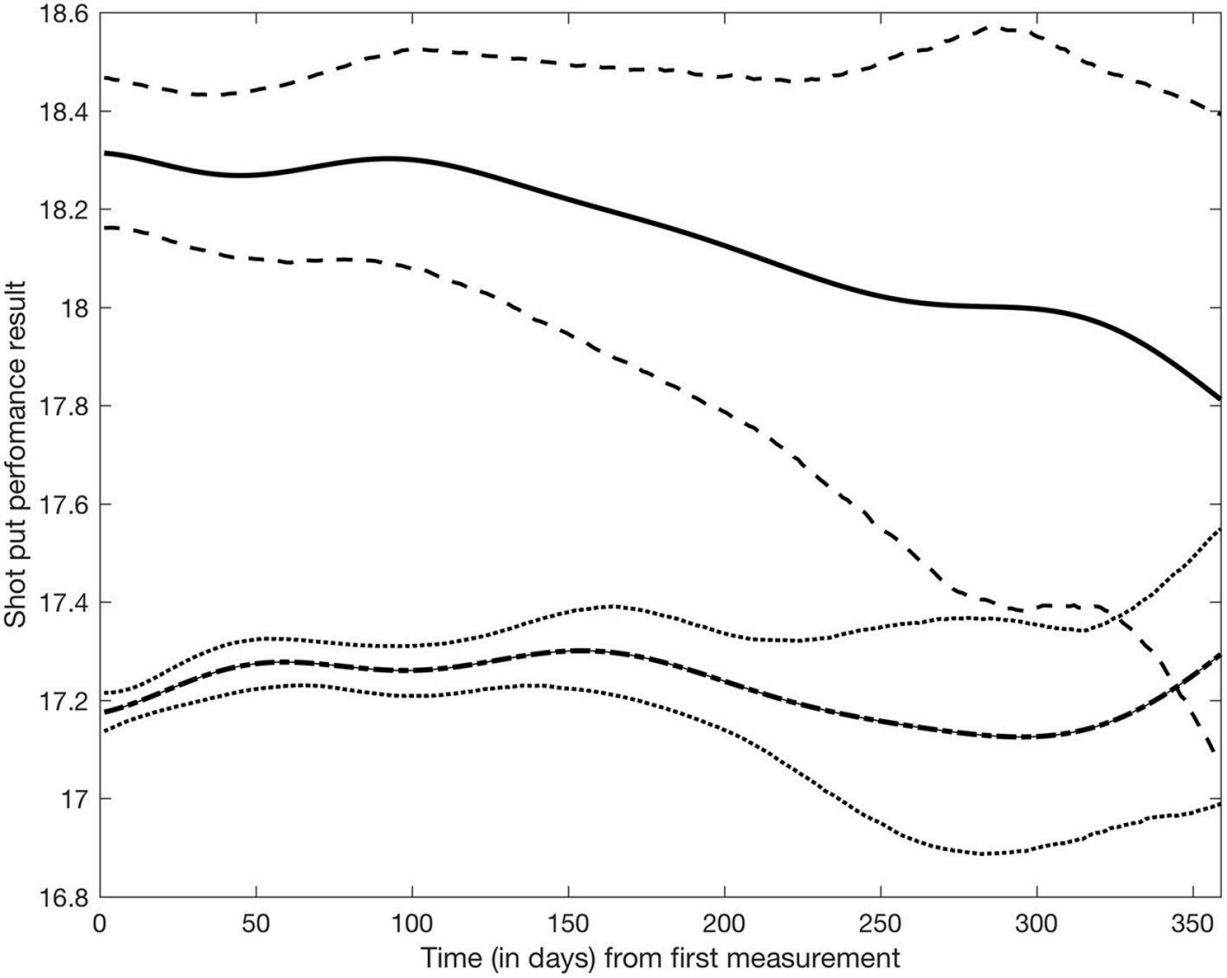
Shot put performance between “non-doped” and “doped” populations. Solid line represents the mean for the “doped” population, with dashed line illustrating the 95% credible performance bounds. Bolded dashed line represents the mean for the “non-doped" population, with dotted lines representing the 95% credible performance bounds.

**Figure 5:**
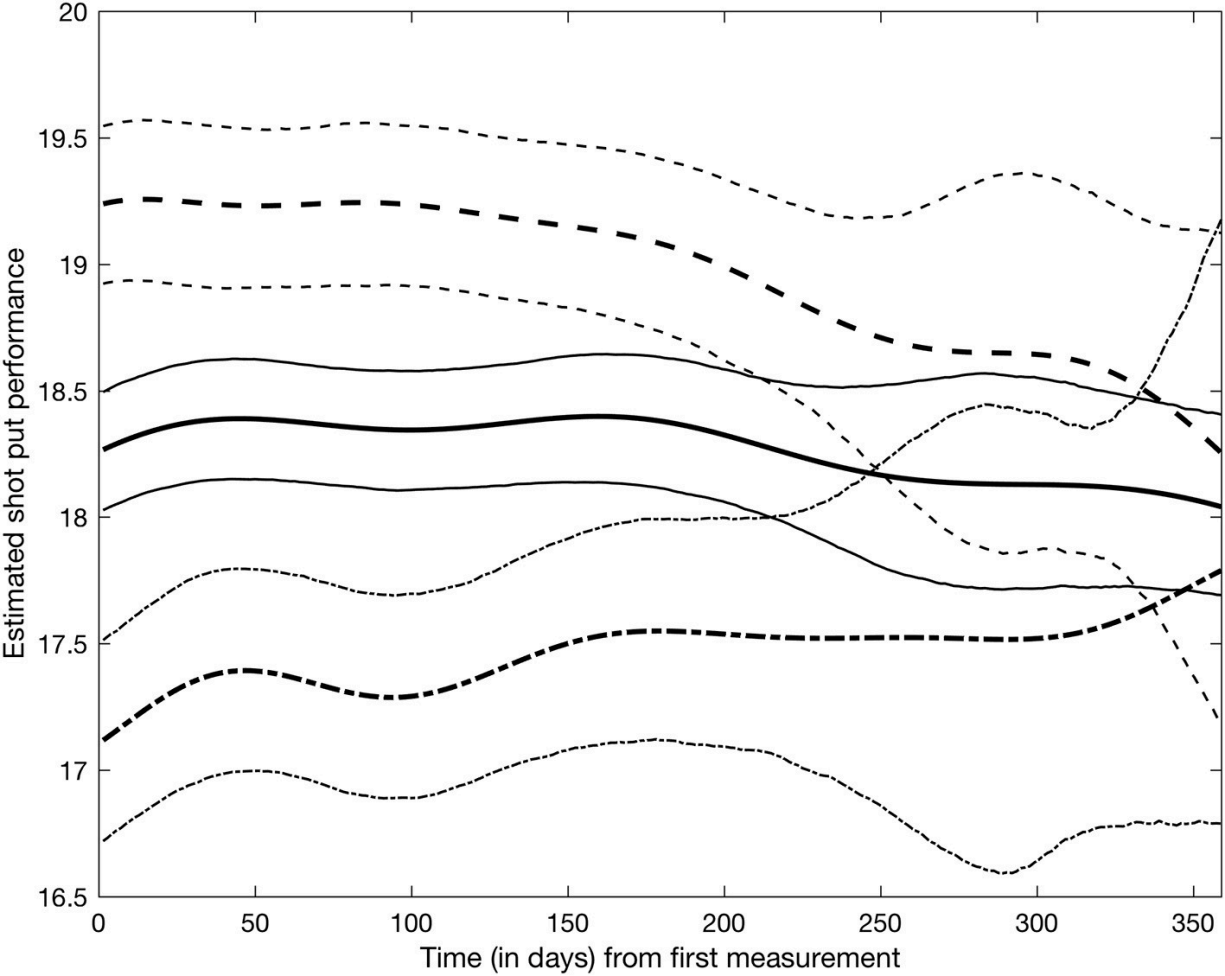
Shotput performance trajectories for a 30 years old male athlete (dash line), a 23 years old male athlete (solid line), and a 15 years old male athlete (dash-dot) along with their 95% credible intervals.

Figure 4 shows the average shot put performance function estimates for doping and non-doping athlete groups. Specifically, the solid and dash-dot lines in Figure 4 were obtained by averaging the basis function coefficients for athletes belonging to either group, ${\hat{f}}_{g}\left(t\right)=\text{b}{\left(t\right)}^{⊤}{\hat{\theta }}_{g}$, where ${\hat{\theta }}_{g}=\frac{1}{\text{Card}\left(g\right)}\underset{i\in g}{\sum }{\hat{\theta }}_{i}$ and Card(*g*) is the cardinality of group *g*. It appears that as a group, doped athletes achieve higher shot put results than non-doped athletes, though credible intervals expand and overlap at the end of the observation period due to lack of data in the later part of the year. Within this model, it is also possible to study the effect of covariates on shot put performance. Figure 5 shows the estimated trajectories ${\hat{f}}_{i}$ for a 30 years old male athlete (dash line), a 23 years old male athlete (solid line), and a 15 years old male athlete (dash-dot line), along with 95% credible intervals. It appears age has an impact on shot put performance, with more experienced athletes outperforming their younger counterparts. No significant conclusions can be made at the end of the year (after day 200) given that credible bands are overlapping. This is again due to the sparsity of the data, thus increased uncertainty.

**Figure 6:**
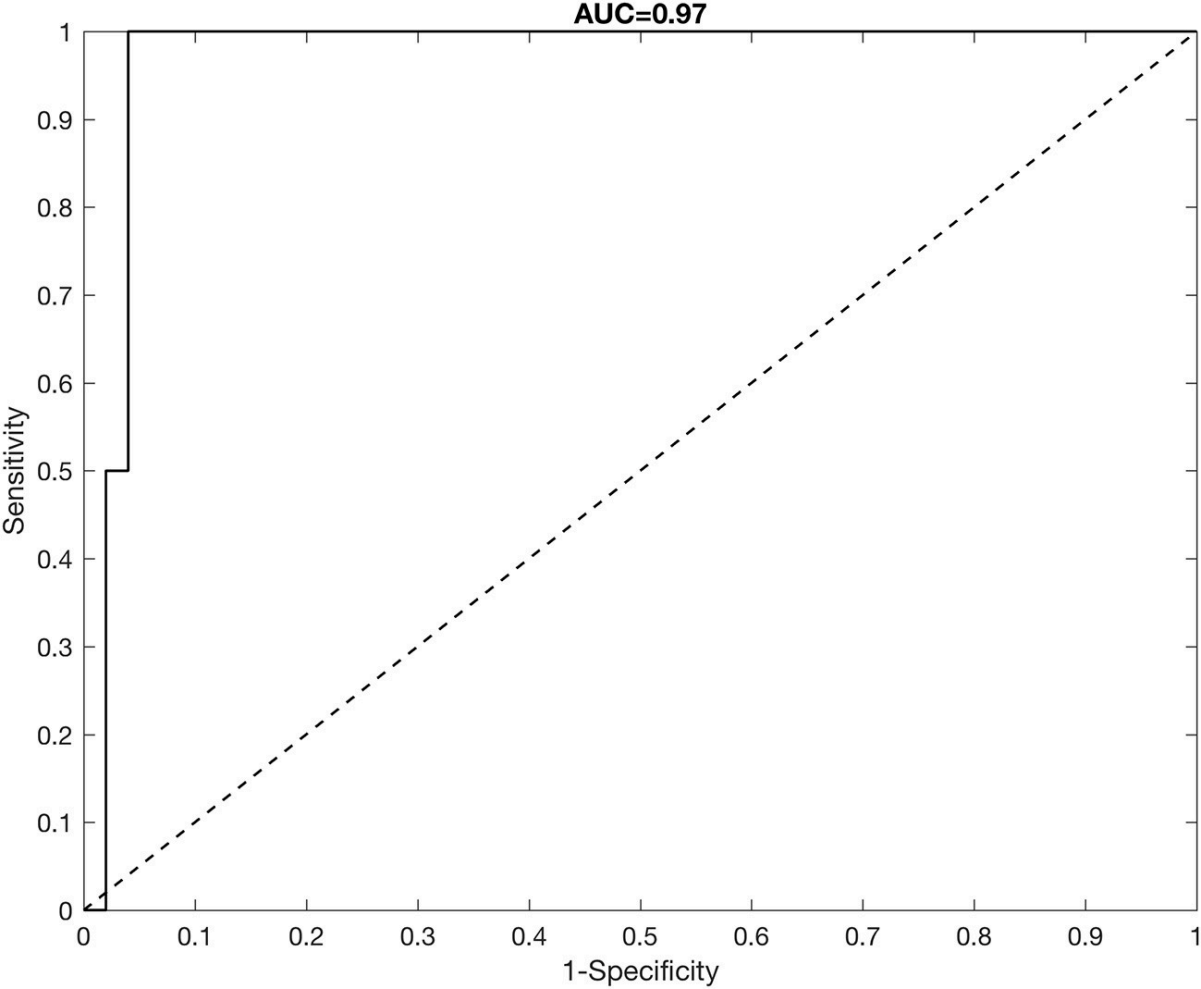
ROC curve analysis for correct classification of doping status for athletes in the test set.

To assess the predictive performance of our model, we split the data into a training set of 300 athletes and a test set of 52 athletes. Training and test sets were generated to maintain the same proportion of doped/non-doped athletes in the sample, thus 2 athletes in the test set (3.69% of 52) were randomly chosen from the doped athletes in the sample. All remaining athletes in the test set were randomly chosen from non-doped athletes in the sample. The complete data (shot put performance and doping status) were retained for the training set when running the analysis, whereas the doping status of athletes in test set was held out and predicted. Figure 6 shows the ROC curve for correct classification of doping status for subjects in the test set. We also report the Area Under the Curve (AUC), with the closer this value to 1, the better the predictive performance. The model shows very good predictive performance, but we underline this result is mostly driven by the correct classification of supposed non-doped athletes as “non-doped,” rather than the classification of the two doped athletes in the test set as “doped” athletes. A discussion is provided in the next section.

## 5. Discussion

This study has proposed a Bayesian latent factor regression model for the analysis of performance data used in an anti-doping context. Following (Montagna et al., 2012), we include a high-dimensional set of pre-specified basis functions, while allowing automatic shrinkage and effective removal of basis coefficients not needed to characterize any of the athlete-specific functions. Further, we accommodate joint modeling of a functional predictor, the elite shot put performance trajectory, with a binary response, the doping status, within a framework of scalar-on-function regression. While the motivation of our work comes from the analysis of shot put performance data, the methodology presented in this paper is widely applicable to the analysis of performance data collected in all so-called centimeter-gram-second sports.

The model achieves good predictive performance of doping status, with a high AUC. However, the model prediction is driven by the correct classification of non-doped athletes, thus the error in sensitivity we observe is due to the misclassification of the doped athletes as non-doped. For example, if we classified as “doped” all athletes having ${\hat{p}}_{i}>0.50$ (the random classification rate), none of the truly doped athletes in the test set would be correctly classified as doped. We observed this misclassification problem not only when analysing data limited to 1 year, but also when analysing all available shot put performance data. There could be several reasons that lead to the misclassification. The number of doped athletes is very small compared to size of the sample (13 doped athletes out of 352 subjects), thus it is possible that the signal is too low for the model to be able to detect the doping-status of an athlete. In particular, (Montagna et al., 2012, 2018) show the model has good classification performance of any group in simulation studies with higher signal-to-noise ratio and more balanced representation of different groups in the sample. It might also be possible that some athletes denoted as “clean” in the sample are in fact “doped,” but were never caught through traditional testing methods.

Regardless of potential issues with the data, the model in its current formulation suffers from some limitations. The Bayesian latent factor regression methodology was originally developed for very sparse longitudinal data (Montagna et al., 2012) with the purpose of capturing a global trend in subject-specific trajectories. Instead, the shot put dataset has measurements often collected just a few days apart from each other on each athlete, and for a potentially long number of years. Thus, our dataset shows more local, short-range variability, which the current version of the model cannot adequately represent. Further, the model does not currently accommodate longitudinal covariates potentially affecting performance, for example, as shown in Figure 5, athlete aging has a clear demonstrable effect on shot put throwing distance. Moreover, factors such as individual athlete seasonal training and competition patterns will affect their individual competition results, independently of any doping related effect and so must be accounted for in any longitudinal model of performance. Nonetheless, our current model appears to be able to detect differences between the two populations in the early phase of the competitive year, with about 1 meter difference in performance between doped and non-doped athletes (see Figure 4). Therefore, this retrospective data modeling provides an indication that the effects of doping can be identified from longitudinal athlete performance profiles, and that it could potentially be used as a tool to estimate theoretical future credible performances for a given athlete. The ultimate application of this type of modeling approach would be that when the projected credible level of performance is exceeded by an athlete (e.g., Figure 3—athlete 53 and 281 data points above the 95% credible interval), they would be identified for target testing via the ABP system. Thus, an athlete performance passport will potentially improve the effectiveness of in-competition anti-doping testing, where currently only placed athletes and a number of randomly selected others, are subjected to scrutiny.

While the above has to be considered as a preliminary analysis affected by some limitations, the model is still credible and might represent a useful tool for longitudinal tracking of athlete performance in order to discriminate expected from unexpected or disproportionate increases. Further research is required in order to increase the structure of the model by incorporating more covariates (e.g., training, maturation, seasonal variation) in order to increase its accuracy and sensitivity, and thus better fit the shot put data.

## Author contributions

JH conceptualized the idea; SM developed the methods and conducted the analysis; JH and SM drafted, read and approved the manuscript.

### Conflict of interest statement

The authors declare that the research was conducted in the absence of any commercial or financial relationships that could be construed as a potential conflict of interest.
